# ATPIF1 maintains normal mitochondrial structure which is impaired by CCM3 deficiency in endothelial cells

**DOI:** 10.1186/s13578-020-00514-z

**Published:** 2021-01-09

**Authors:** Kang Wang, Haixuan Chen, Zhongyang Zhou, Haifeng Zhang, Huanjiao Jenny Zhou, Wang Min

**Affiliations:** 1 Center for Translational Medicine, The First Affiliated Hospital, Sun Yat-Sen University, Guangzhou, 510080 China; 2 Interdepartmental Program in Vascular Biology and Therapeutics, Department of Pathology, Yale University School of Medicine, New Haven, CT 06520 USA

**Keywords:** CCM3, ATPIF1, CRISPR-Cas9, Mitophagy, KLF4

## Abstract

**Background:**

Numerous signaling pathways have been demonstrated experimentally to affect the pathogenesis of cerebral cavernous malformations (CCM), a disease that can be caused by CCM3 deficiency. However, the understanding of the CCM progression is still limited. The objective of the present work was to elucidate the role of CCM3 by RNA-seq screening of CCM3 knockout mice.

**Results:**

We found that ATPIF1 was decreased in siCCM3-treated Human Umbilical Vein Endothelial Cells (HUVECs), and the overexpression of ATPIF1 attenuated the changes in cell proliferation, adhesion and migration caused by siCCM3. The probable mechanism involved the conserved ATP concentration in mitochondria and the elongated morphology of the organelles. By using the CRISPR-cas9 system, we generated CCM3-KO Endothelial Progenitor Cells (EPCs) and found that the knockout of CCM3 destroyed the morphology of mitochondria, impaired the mitochondrial membrane potential and increased mitophagy. Overexpression of ATPIF1 contributed to the maintenance of normal structure of mitochondria, inhibiting activation of mitophagy and other signaling proteins (e.g., KLF4 and Tie2). The expression of KLF4 returned to normal in CCM3-KO EPCs after 2 days of re-overexpression of CCM3, but not other signaling proteins.

**Conclusion:**

ATPIF1 maintains the normal structure of mitochondria, inhibiting the activation of mitophagy and other signaling pathway in endothelial cells. Loss of CCM3 leads to the destruction of mitochondria and activation of signaling pathways, which can be regulated by KLF4.

## Background

Cerebral cavernous malformations (CCMs) are characterized by enlarged and irregular small blood vessels that lack pericytes and elastic tissue. These vessels have a thin wall, are leaky, and lack sub-endothelial support. CCMs affect 0.5% of the population and are primarily found in the vasculature of the central nervous system, where they trigger cerebral hemorrhage, seizures, and stroke [1]. Currently, no effective pharmacotherapy is available for CCM, and surgical resection is the only treatment. However, in some cases, CCM lesions are located deep in the brain tissue, making surgery impossible. Therefore, the identification of therapeutic targets for CCM is of great importance. Novel pharmacological strategies are particularly needed to treat patients with severe symptomatic disease due to inoperable or multiple lesions, and prevent de novo formation of CCM lesions and disease progression in susceptible individuals [2].

CCMs are associated with loss-of-function mutation in any of the three CCM genes: *CCM1* (*KRIT1*) [3], *CCM2* [4], and *CCM3* (*PDCD10*) [5]. Importantly, mutations in *CCM3* are frequently associated with higher risk of early-onset cerebral hemorrhage and more severe form of the disease [6, 7]. These properties suggest a separate or unique role of CCM3, distinct from CCM1 and CCM2. CCM3 has been shown to regulate CCMs pathogenesis by participating in different cellular functions and signaling pathways, including the proliferation, migration, adhesion of cells and angiogenesis. Loss of CCM3 activates ERK1/2, which, in turn, induces serine-phosphorylation and degradation of cortactin, affecting the stability of tight junction complexes [8]. The knockdown of CCM3 also increases the phosphorylation of Myosin Light Chain (MLC), suggesting the activation of the RhoA-ROCK signaling. This effect is related to the CCM3-STK25-ERM pathway [9]. ROCK inhibitors rescue the defects of cell migration, invasion, and three-dimensional tube formation caused by knockdown of CCM3. Therefore, targeting RhoA-ROCK signaling may represent a potential therapeutic strategy for CCMs. Loss of CCM3 also induces exocytosis of angiopoietin 2 and activates the ANGPT2-Tie2 pathway, thus inducing angiogenesis in endothelial cells [10]. Numerous cellular activities and signaling pathways have been demonstrated to regulate CCMs progression, but no consensus has been reached regarding its fundamental mechanism. In fact, mutually exclusive hypotheses have been proposed to explain the exact function of CCM3 in cell survival [11, 12] and the association between the Endothelial to Mesenchymal Transition (EndMT) and the loss of CCM genes [13, 14]. Thus, additional research is needed to understand the function of CCM3 in the pathogenesis of CCMs.

Besides producing ATP, mitochondria in endothelial cells play an important role in regulating signaling responses to environmental cues [15]. Hypoxia increases the level of VEGF, which enhances mitochondrial biogenesis through Akt-dependent signaling, resulting in vascular sprouting [16]. High glucose concentration triggers endothelial injury by increasing mitochondrial fission and elevating the production of reactive oxygen species (ROS), leading to dysfunction and apoptosis of endothelial cells [17]. Mitochondria are highly dynamic organelles, and their fusion and fission are associated with angiogenesis [18, 19]. Mitochondrial impairment alters the formation of ROS and membrane potential, resulting in endothelial dysfunction or apoptosis. ATPIF1 is a mitochondria-localized protein encoded by nuclear DNA. Upon binding to α and β subunits of the F1 complex, ATPIF1 inhibits the hydrolysis of ATP by ATP synthase [20]. Several studies suggested that ATPIF1 regulates the morphology of mitochondria and the cellular survival pathway [21–23]. Although mitochondria are essential for the function of endothelial cells, the understanding of the effect of CCM3 on mitochondria is rather limited. Here, we demonstrate that the loss of CCM3 leads to the destruction of mitochondria and modifies signaling pathways, while overexpression of ATPIF1 attenuates these alterations.

## Results

### Changes in gene expression in endothelial cells after CCM3 knockdown

To comprehensively evaluate the effect of *CCM3*-ablation in endothelial cells, we compared the levels of mRNAs in endothelial cells of cerebellar microvasculature obtained from wild-type (WT) (*Ccm3*^fl/fl^) and *Mfsd2a-CreERT2*; *Ccm3*^fl/fl^ (*Ccm3*^ECKO^) mice [10]. Ninety-two genes with significant differential expression were subjected to the DAVID Gene Ontology Biological Process (GOBP) analysis. The results indicated that the significantly enriched (p < 0.005) pathways comprised mitochondria-related oxidative phosphorylation, cell junction and angiogenesis (Table 1, Additional file 1: Fig. S1).

**Table 1:** Gene Ontology Biological Process analysis of for *Ccm3*^ECKO^ mice RNA-Seq results

Pathway term	Count	*p*-value	Genes
Hydrogen ion transmembrane transport	11	2.50E−13	NDUFA4, COX7A2, UQCRH, COX8A, COX7C, COX6B1, COX6A1, COX5A, UQCRQ, COX6C, UQCRB
Mitochondrial electron transport, cytochrome c to oxygen	8	3.14E−12	NDUFA4, COX8A, COX7C, COX6B1, COX6A1, COX5A, COX5B, COX6C
Mitochondrial ATP synthesis coupled proton transport	7	4.95E−10	ATP5E, ATP5J2, ATP5L, ATP5O, ATP5G2, ATP5G1, COX5B
ATP biosynthetic process	6	2.35E−07	ATP5E, ATP5J2, ATP5L, ATP5O, ATP5G2, ATP5G1
Mitochondrial electron transport, NADH to ubiquinone	6	3.50E−06	NDUFA4, NDUFB11, NDUFB5, NDUFA8, NDUFC2, NDUFA13
Generation of precursor metabolites and energy	5	1.17E−04	COX8A, COX7C, COX6A1, ATPIF1, COX6C
ATP synthesis coupled proton transport	4	1.50E−04	ATP5L, ATP5O, ATP5G2, ATP5G1
Mitochondrial respiratory chain complex I assembly	5	2.30E−04	NDUFB11, NDUFB5, NDUFA8, NDUFC2, NDUFA13
Cell–cell adhesion	8	3.07E−04	CCT8, WASF2, S100A11, RANBP1, EEF1D, PARK7, AHNAK, ANXA2
Mitochondrial electron transport, ubiquinol to cytochrome c	3	0.002261	UQCRH, UQCRQ, UQCRB
Angiogenesis	6	0.004235	PTPRB, CAV1, CLIC4, WASF2, ATPIF1, ANXA2

**Fig. 1:**
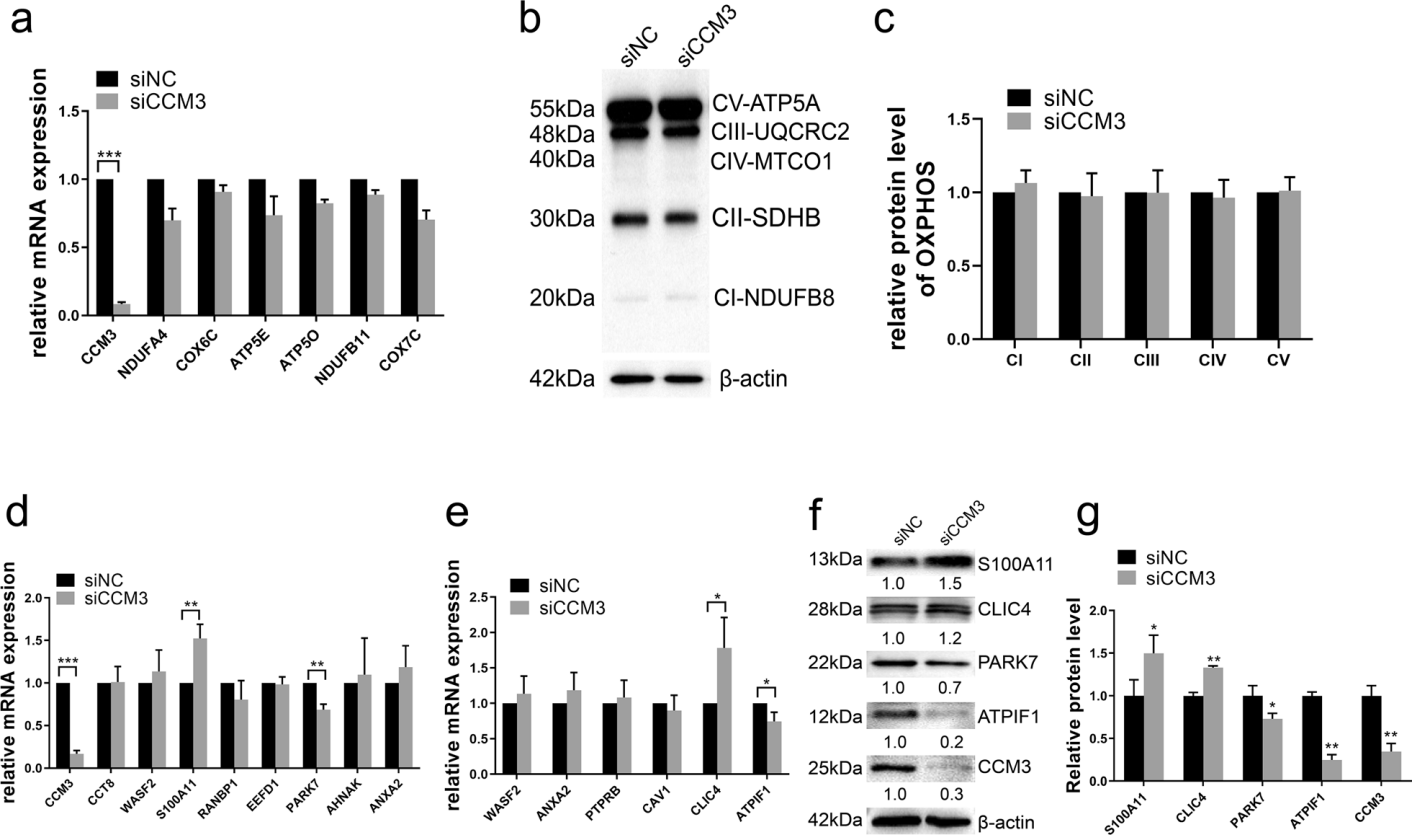
Identification of new markers in endothelial cells after CCM3 knockdown. **a** mRNA changes of electron transport chain and OXPHOS genes from RNA-seq in HUVECs after siNC and siCCM3 treatment by qRT-PCR. **b-c** protein changes of OXPHOS in HUVECs by Western Blotting (**b**), and the quantification of each band (**c**), β-actin was blotted as internal control. mRNA changes of cell–cell adhesion (**d**) and angiogenesis (**e**) pathway genes from RNA-seq results in HUVECs, the significantly changed genes were also examined by Western Blotting (**f**) and quantified (**g**). qRT-PCR results were expressed as mean ± SD (n = 3). *p < 0.05, **p < 0.01, ***p < 0.001

Previous studies have documented that cell-to-cell junctions and angiogenesis are the essential processes involved in the pathogenesis of CCM. However little research was focused on the changes in mitochondria. This limitation is surprising since several investigations have shown that CCM proteins protect cells against oxidative stress, particularly in the form of ROS [11]. To further confirm the changes in mRNA expression in human endothelial cells, the differentially expressed genes were analyzed in HUVECs after CCM3 knockdown. We found that the mRNA levels of most genes related to mitochondrial ATP synthesis were decreased after exposure of cells to siCCM3 (Fig. 1a), but the protein levels remained essentially constant (Fig. 1b, c). Since endothelial cells generate ATP mostly through glycolysis [15], we also determined the expression of glycolysis-related genes after siCCM3 treatment and found that most of them were upregulated (Additional file 2: Fig. S2a). In fact, CCM3 knockout had a similar effect on oxidative phosphorylation and glycolysis in EPCs (Additional file 2: Fig. S2b, d). Among genes related to cell-to-cell adhesion and angiogenesis, expression of four of them, *S100A11*, *CLIC4*, *PARK7*, and *ATPIF1*, changed significantly at the mRNA level (Fig. 1d, e). Western Blotting demonstrated that the siCCM3-induced changes in protein levels were most apparent for *S100A11* (increased) and *ATPIF1* (decreased) (Fig. 1f, g). These findings indicate a more powerful impact of CCM3 on S100A11 and ATPIF1.

Total 92 genes with significant differential expression were subjected to DAVID GOBP analysis. Each enriched GOBP cluster with *p* < 0.005, FDR q < 0.1, overlap cutoff > 0.5.

### Overexpression of ATPIF1 restores cell proliferation, junctions and migration which was impaired by CCM3-knockdown

**Fig. 2:**
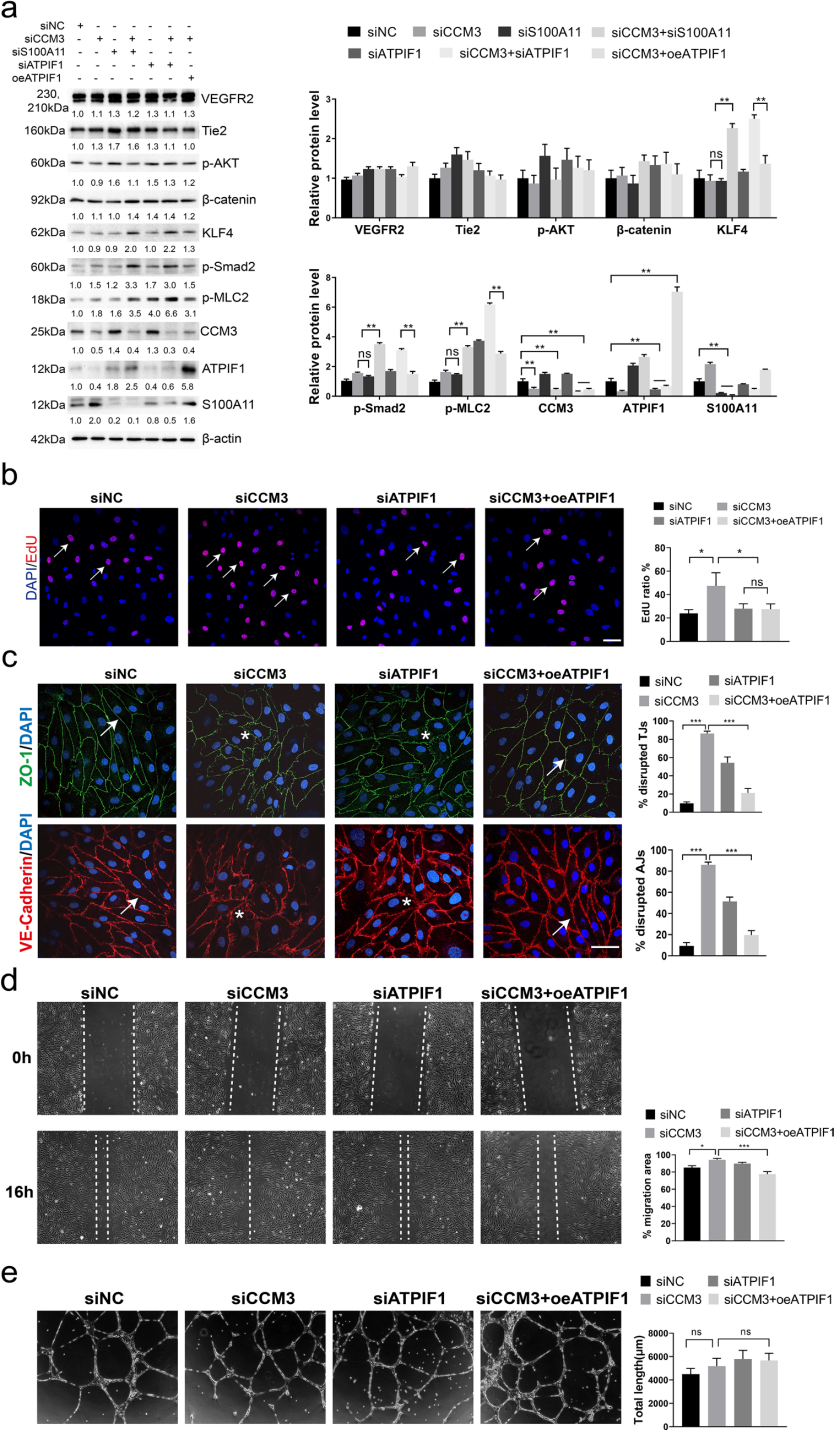
Overexpression of ATPIF1 restore cell proliferation, junction and migration which was impaired by CCM3-knockdown. **a** The changes of already proved signaling pathways in HUVECs after siCCM3 with siS100A11 or oeATPIF1 treatment. **b** EdU staining in HUVECs after siRNA or overexpression treatment, scale bar, 50 μm. **c** Immunofluorescence images about tight junction and adherent junction changes in HDMECs after siCCM3 with oeATPIF1 treatment, and the percentages of disrupted tight junctions (TJs) and adherent junctions (AJs) (3 random microscope fields were used for quantification). HUVECs monolayer migration assay (**d**) and Matrigel based tube formation assay (**e**) after siCCM3 and oeATPIF1 treatment, migration area or total branches length was measured by Image J (n = 3). *p < 0.05, **p < 0.01, ***p < 0.001. Scale bar, 50 μm

Many signaling pathways, such as the angiopoietin 2-Tie2 pathway [10], MEKK3-KLF4 pathway [13], and the ROCK pathway [24, 25], are associated with CCM3-deficiency. Therefore, we evaluated the changes in signaling pathways after simultaneous knockdown of S100A11 and CCM3 by the combination of siS100A11 and siCCM3, or infect siCCM3-treated HUVECs with pLEX-ATPIF1 (the CDS of ATPIF1 was inserted in the LentiORF pLEX-MCS vector) lentivirus for 48 h. The Western Blotting results indicated that siS100A11 does not restore the previously determined signaling after the siCCM3 treatment, siS100A11 even activates KLF4, p-Smad2 and p-MLC2 with siCCM3 in HUVECs (Fig. 2a). But overexpression of ATPIF1 apparently restored the level of p-Smad2 and p-MLC2 (Fig. 2a). Therefore, we presumed that the ATPIF1 might represent a more effective target regulating CCM3 related signaling pathways.

Earlier studies have proved that the loss of CCM3 leads to the degradation of cell junctions, increased cell proliferation, migration [10, 26], tube formation and angiogenesis [27, 28]. Thus, to examine the effect of ATPIF1 on the structure and function of endothelial cells, we performed immunofluorescence staining of HUVECs and human dermal microvascular endothelial cells (HDMEC) after the administration of siCCM3 or a combination of siCCM3 and overexpression of ATPIF1 (oeATPIF1). EdU staining results indicated that siCCM3 treatment activates cell proliferation, but siATPIF1 treatment doesn’t have significant effect on cell proliferation, and overexpression of ATPIF1 inhibits siCCM3 related cell proliferation activation (Fig. 2b). Treatment with siCCM3 disrupted the tight junctions (ZO-1) and adherent junctions (VE-cadherin). A similar phenotype was obtained with siATPIF1, but the combination of siCCM3 and oeATPIF1 restored the cell junctions to a significant extent (Fig. 2c). Additionally, the migration assay showed a similar effect, siCCM3 and siATPIF1 increased the migration rate when compared to the negative control (siNC), and the combination of siCCM3 and oeATPIF1 reduced the migration rate (Fig. 2d). However, the latter treatment did not affect the ability of cells to form tubes (Fig. 2e). This lack of effect was likely because tube formation depends on the combination of gene expression and epigenetic or local microenvironmental factors [12]. Together, these data suggest that overexpression of ATPIF1 can attenuate abnormalities in cell proliferation, junctions and migration caused by siCCM3, and the effect on cell junction and migration may be mediated by regulating the ROCK-related pathway via p-MLC2.

### Overexpression of ATPIF1 maintains the linear mitochondrial phenotype

**Fig. 3:**
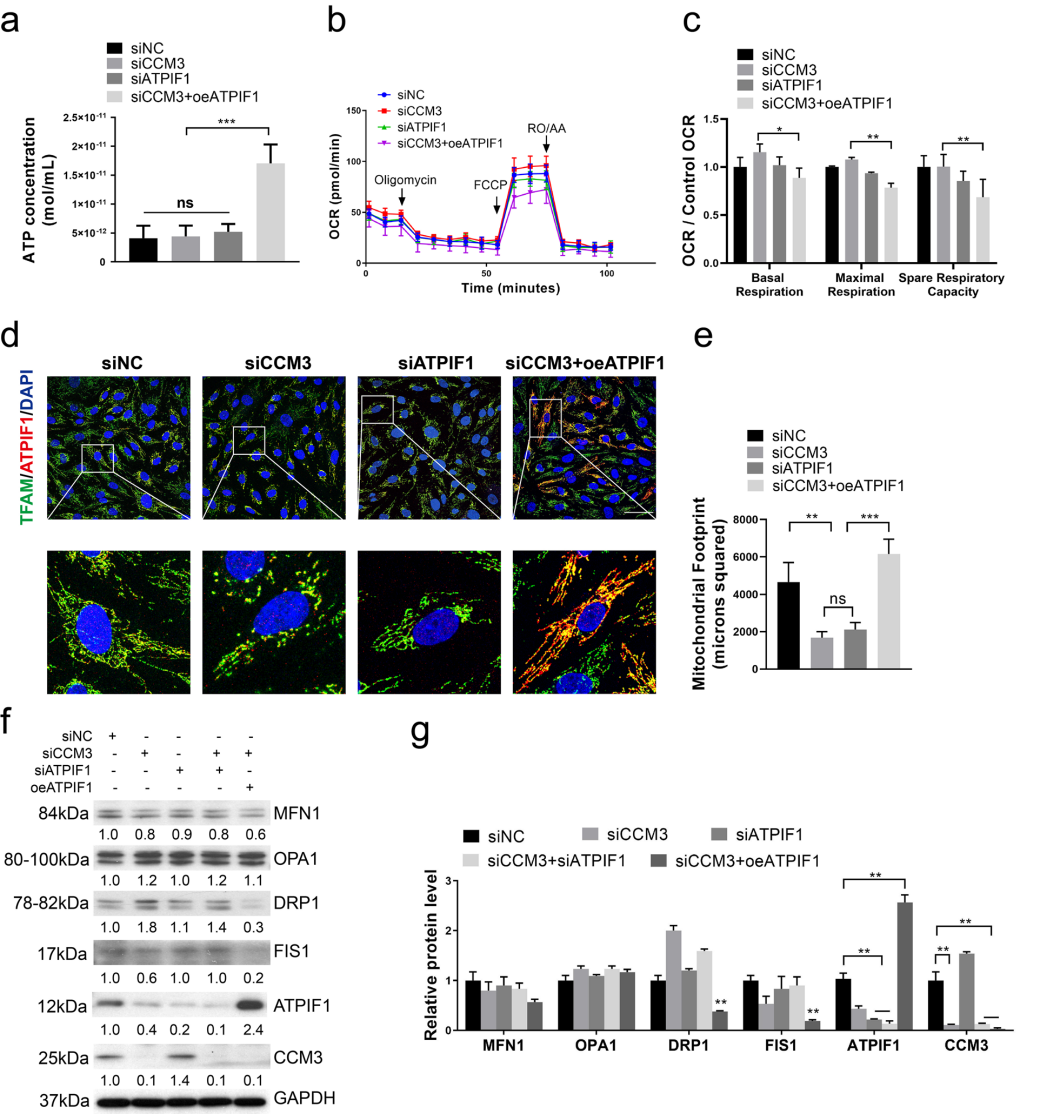
Overexpression of ATPIF1 maintain the linear mitochondrial phenotype. **a** mitochondrial ATP concentration in HUVECs after siCCM3 and oeATPIF1 treatment, n = 3. **b** mitochondrial mito-stress assay in HUVECs after treatment by seahorse, cells were challenged with oligomycin (1 μM), FCCP (1 μM) and RO/AA(1 μM). **c** quantification of basal respiration, maximal respiration and spare respiration capacity in HUVECs after treatment, n = 6. **d** Immunofluorescence images and quantification (**e**) of mitochondria in HUVECs after treatment. **f** Western Blotting about mitochondrial fusion and fission proteins in HUVECs after treatment and quantification (**g**). *p < 0.05, **p < 0.01, ***p < 0.001. Scale bar, 50 μm

**Fig. 4:**
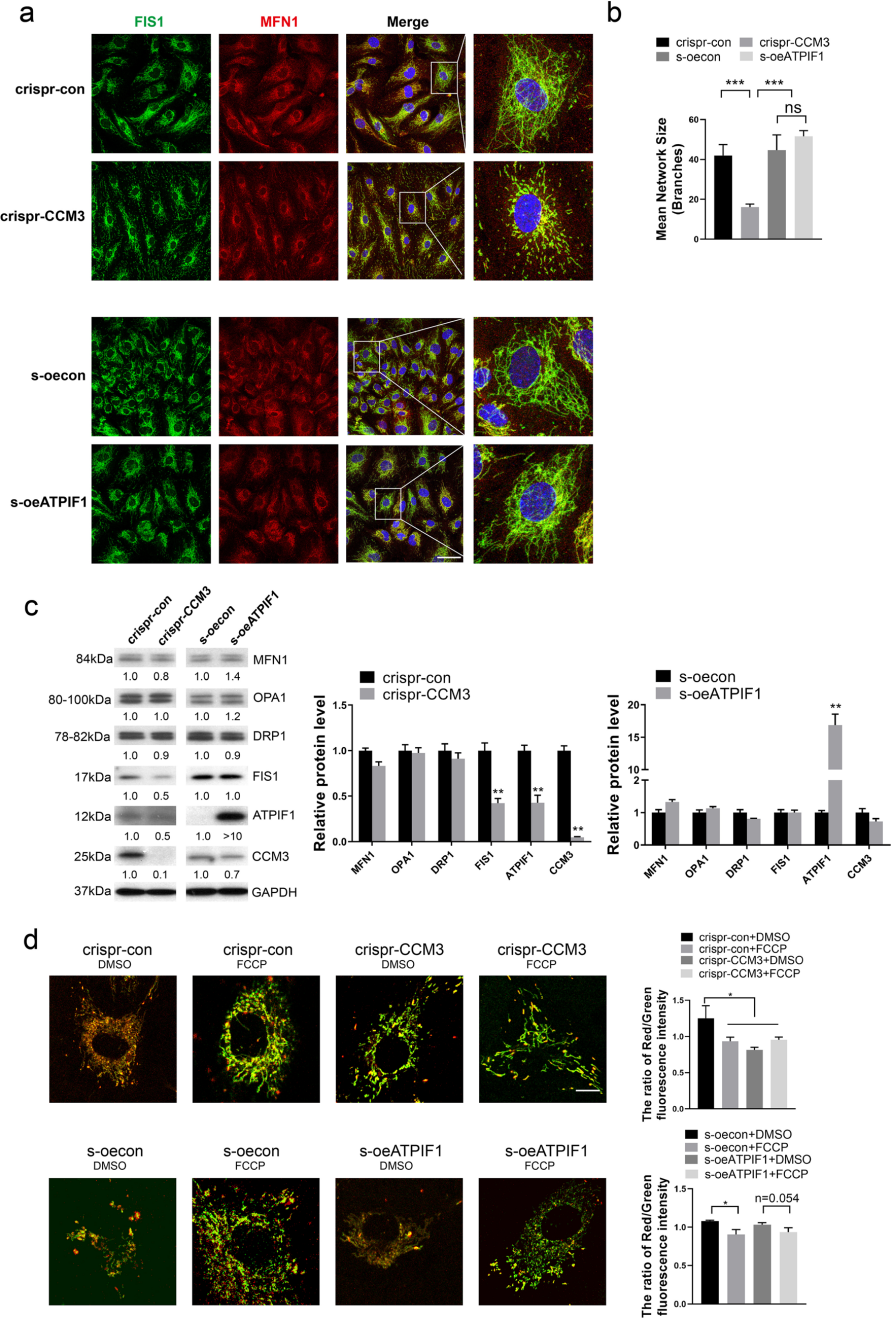
Knockout CCM3 compromised mitochondrial membrane potential. **a** Immunofluorescence images and quantification (**b**) of mitochondria in selected CRISPR-CCM3 EPCs and s-oeATPIF1 EPCs, scale bar, 50 μm. **c** mitochondrial fusion and fission markers in selected EPCs. **d** mitochondrial membrane potential in selected EPCs by JC-1 assay, cells were treated with FCCP (10 μM) for 2 h, DMSO was used as negative control. Mean fluorescence intensity (MFI) was measured by Image J, n = 3, *p < 0.05, **p < 0.01, scale bar, 10 μm

ATPIF1 binds to the α and β subunits of ATPase inhibiting ATP hydrolysis. To test the hypothesis that ATPIF1 can preserve the intracellular ATP content, we examined the mitochondrial ATP content in HUVECs. Overexpression of ATPIF1 resulted in a higher ATP concentration than treatment with siRNA (Fig. 3a), this result means ATPIF1 prevent the mitochondria from wasting ATP by inhibiting ATPase activity. To further explore the impact of siCCM3, siATPIF1 and overexpression of ATPIF1 on cell metabolism, metabolic profiling was performed using the Cell Mito stress test kit. The oxygen consumption rate (OCR) of mitochondria was measured in siRNA or overexpression treated HUVECs with the Seahorse XF instrument. The pre-treated HUVECs were orderly challenged with oligomycin to block ATP synthesis (complex V), carbonyl cyanide 4-trifluoromethoxy-phenylhydrazone (FCCP) to uncouple the mitochondrial inner membrane (MIM), and the combination of rotenone (RO) and antimycin A (AA) to inhibit mitochondrial respiration (complex I/III). The results suggested that siCCM3 and siATPIF1 did not impact the OCR significantly. However, the treatment with siCCM3 and oeATPIF1 combined decreased basal respiration, maximal respiration, and spare respiratory capacity (Fig. 3b, c). This effect may be related to the interaction between ATPIF1 and ATP synthase, since ATPIF1 can block ATP synthase F1 subunit, inhibiting the normal rotation of F1 subunit which is essential for ATP synthesis during oxidative phosphorylation. The results from Fig. 3a–c led to a hypothesis, which means the hydrolysis of ATP in siCCM3 HUVECs was increased significantly after ΔΨm was compromised (the change of ΔΨm was demonstrated in Fig. 4d), the ATP content preserved by overexpression of ATPIF1 was more than the effect of inhibition of ATP synthase.

We have also found that in addition to affecting mitochondrial function, ATPIF1 affects their morphology. The immunofluorescence images revealed significant differences between cells treated with siCCM3 and oeATPIF1. The treatment with siCCM3 and siATPIF1 shortened the total length of mitochondria, and mitochondria assumed a morphology of small spheres or short rods (Fig. 3d, e). In contrast, mitochondria in cells exposed to oeATPIF1 became elongated (Fig. 3d, e), consistent with mitochondrial fusion. These findings implied the modifications of mitochondrial fusion and fission dynamic, but the Western Blotting results did not provide the evidence of changes in the expression of proteins regulating mitochondrial fusion, MFN1 and OPA1, after siRNA or overexpression treatment (Fig. 3f, g). This discrepancy might have been caused by the modulation of activity or localization of these proteins [22]. However, fission markers, DRP1 and FIS1, were both decreased after the treatment with the combination of siCCM3 and oeATPIF1 (Fig. 3f, g), indicating that mitochondrial fission was inhibited by oeATPIF1. We also found that oeATPIF1 increased oxidative phosphorylation and glycolysis to some extent (Additional file 2: Fig. S2c, e). These results suggest that the overexpression of ATPIF1 preserves the intracellular concentration of ATP concentration and maintains the elongated morphology of mitochondria.

### Knockout of CCM3 compromises mitochondrial membrane potential

Because siRNA treatment constitutes a type of a transient stimulus, efficiency of siRNA may decrease with cell proliferation, the siRNA treatment may not accurately reflect the changes taking place in *vivo*. To address this issue, we have constructed CRISPR-hSpCas9 [29] lentivirus targeting CCM3 to knockout CCM3 in EPCs (CRISPR-CCM3). The cells with effective knockout were selected using puromycin. These cells resemble more closely the in vivo condition because they experience the loss of CCM3 for at least 5 days, and maintain a more permanent loss of CCM3 than cells treated with siCCM3. For comparison, in other studies, mice retina was collected at 5 days after tamoxifen injection in *vivo* to determine the effect of CCM3 deficiency on angiogenesis [10]. We also constructed the EPCs with stably overexpressed ATPIF1 (s-oeATPIF1) by using pLEX-ATPIF1 lentivirus and puromycin selection. Empty pLEX-vector was used to construct negative control (s-oecon). The efficiency of knockout or overexpression was confirmed by qRT-PCR, Western Blotting and immunofluorescence (Additional file 3: Fig. S3). Subsequently, the selected cells were stained with MFN1 and FIS1 antibodies. The mitochondria in CRISPR-CCM3 EPCs exhibited a severely disrupted morphology with a much smaller number and fragmented mitochondria (Fig. 4a, b). The protein levels of OPA1, MFN1, and DRP1 were not changed significantly in CRISPR-CCM3 EPCs, while the level of FIS1 was decreased (Fig. 4c), probably due to the lower number of mitochondria. In comparison with negative control, there were no evident changes in morphology or fusion and fission markers in selected oe-ATPIF1 EPCs, this result may suggest ATPIF1 regulates mitochondrial morphology through a pathway other than fission and fusion. Indeed, other study has proved that ATPIF1 increases the cristae number and the length of mitochondria by forming dimeric ATP synthase complexes [21]. Since the significant changes of mitochondrial morphology in CRISPR-CCM3 EPCs suggested functional changes in mitochondria, the ΔΨm was determined by JC-1 assay. The results demonstrated that the knockout of CCM3 decreased ΔΨm to the same extent as the uncoupling factor FCCP, but overexpression of ATPIF1 failed to significantly restore the ΔΨm (Fig. 4d). This lack of effect was probably because ATPIF1 inhibits the hydrolytic activity of ATPase, hence keeps the ΔΨm of EPCs under compromised condition after challenging with FCCP. Together, these findings document that CCM3 has a significant effect on the morphology and function of mitochondria.

### Overexpression ATPIF1 reverses the changes in signaling triggered by CCM3 knockout

**Fig. 5:**
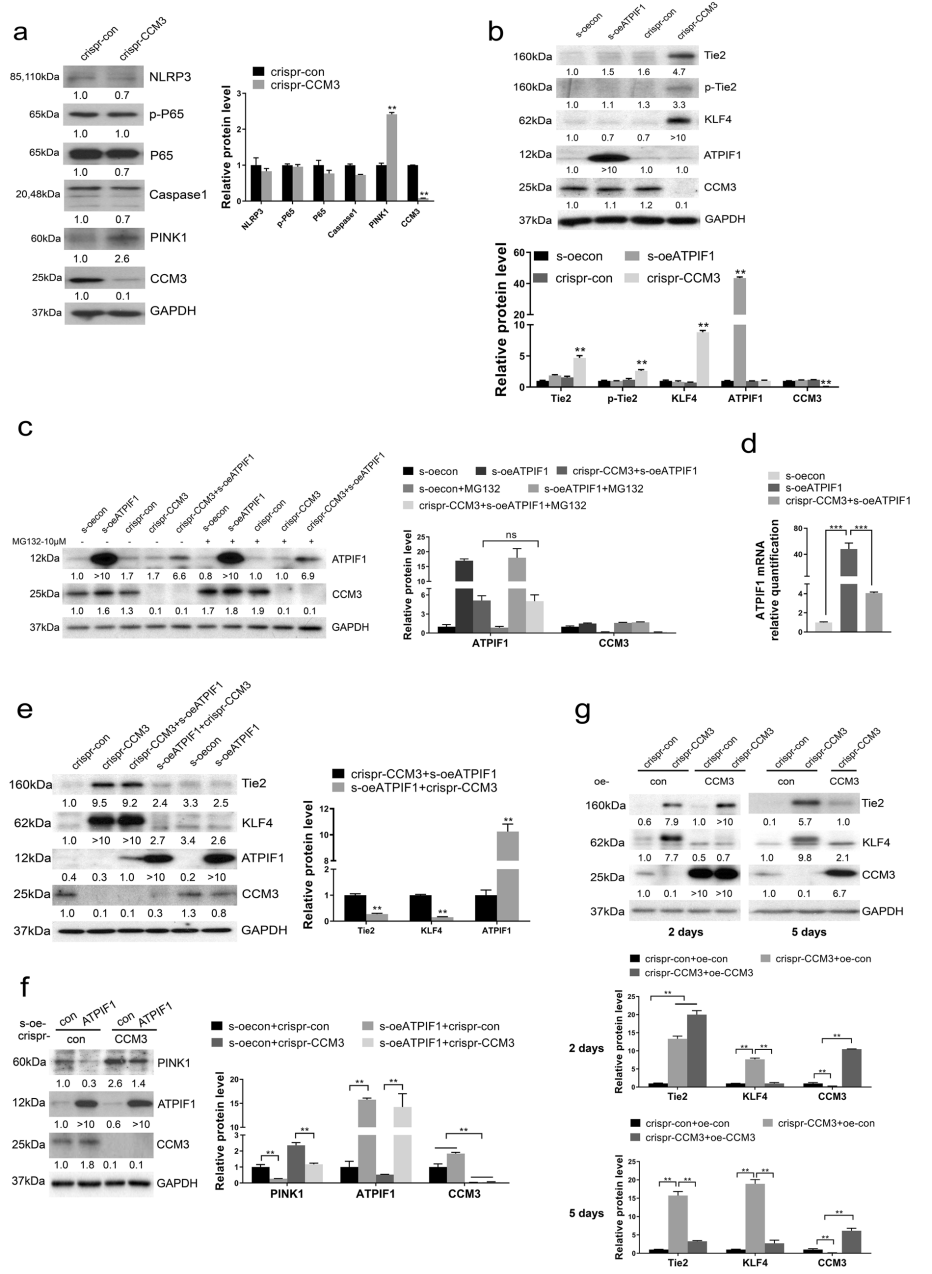
Overexpression ATPIF1 restore the signaling changes caused by CCM3-KO at early stage. **a** change of inflammatory pathway and mitophagy in CRISPR-CCM3 EPCS. **b** change of Tie2, p-Tie2 and KLF4 in selected CRISPR-CCM3 EPCs and s-oeATPIF1 EPCs. **c** MG132 (10 μM) treatment in CRISPR-CCM3, s-oeATPIF1 and CRISPR-CCM3 + s-oeATPIF1 EPCs. **d** ATPIF1 mRNA expression in s-oeATPIF1 and CRISPR-CCM3 + s-oeATPIF1 EPCs, *p < 0.05, **p < 0.01, ***p < 0.001. **e** different changes of Tie2 and KLF4 in CIRPSR-CCM3 + s-oeATPIF1 and s-oeATPIF1 + CRISPR-CCM3 EPCs. **f** the change of PINK1 after s-oeATPIF1 treatment. **g** overexpression CCM3 in CRISPR-CCM3 EPCs for 2 days or 5 days, vector was used as negative control

Dramatic changes in mitochondria suggest the presence of fundamental alterations of certain signaling pathways. To test this possibility, we determined inflammatory pathway, mitophagy and selected CCM3-related signaling pathway in CRISPR-treated and overexpressed EPCs, Western Blotting results showed that CCM3-KO has no evident effect on inflammatory proteins, but increases mitophagy protein PTEN induced putative kinase 1 (PINK1) significantly (Fig. 5a). s-oeATPFI1 had essentially no effect on the analyzed markers, but CRISPR-CCM3 significantly increased well-established markers like KLF4 and Tie2 (Fig. 5b). Tie2 was shown to be associated with the exocytosis of angiopoietin 2 and enhancement of angiogenesis [10], and KLF4 has long been considered as a key factor in the pathogenesis of CCM [13, 30], and it’s also a regulator of mitochondrial homeostasis [43].

We have attempted to overexpress ATPIF1 in EPCs after the CRISPR-CCM3 treatment (the sample labeled as CRISPR-CCM3 + oe-ATPIF1). However, significant overexpression of ATPIF1 was difficult to obtain in CCM3-KO EPCs, although ATPIF1 could be successfully overexpressed in control EPCs (Fig. 5c and Additional file 3: Fig. S3b). We raised the possibility that the knockout of CCM3 destabilize ATPIF1, but the results of the protein degradation assay by MG132 treatment demonstrated that this effect was not related to ubiquitination-mediated degradation (Fig. 5c). Actually, CCM3-KO inhibited the expression of ATPIF1 (Fig. 5d). The effect was also not related to the inhibition of the promoter in the overexpression vector because the re-overexpression of CCM3 was successful (Fig. 5g). Since the probable explanation was that disruption of mitochondria impacts the ATPIF1 protein, we knocked out CCM3 in the s-oeATPIF1 EPCs (the sample labeled as s-oeATPIF1 + CRISPR-CCM3), mitochondria in these cells were intact and signaling pathway proteins were assessed by Western Blotting. The results indicated that the efficiency of knockout and overexpression was adequate, and the levels of signaling markers KLF4 and Tie2 were comparable to the control (Fig. 5e). In contrast, the sample treated with s-oeATPIF1 after CRISPR-CCM3 behaved similarly to the CRISPR-CCM3 sample, i.e., the signaling markers were significantly increased (Fig. 5e). The expression of PINK1 was also decreased after s-oeATPIF1treatment (Fig. 5f), suggesting ATPIF1 plays a role in mitophagy inhibition. We have also found that, after several passages, the s-oeATPIF1 + CRISPR-CCM3 EPCs had morphology and signaling comparable to CRISPR-CCM3 EPCs (data not shown). To ensure that all changes in the morphology and signaling were due to the loss of CCM3 but not to the off-target effects of CRISPR-Cas9, we re-overexpressed CCM3 in CCM3-KO EPCs. The re-overexpression was efficient, and interestingly, only KLF4 was restored after re-overexpression of CCM3 for 2 days, while all other markers were recovered after 5 days (Fig. 5g). Based on this finding, we speculated that KLF4 could be the upstream regulator of the changes in the signaling and mitochondrial morphology. These data suggest that overexpression of ATPIF1 maintained the normal signaling and mitochondrial morphology early after CCM3 knockout, which later led to significant alterations in the mitochondrial phenotype and signaling pathways, and the increased KLF4 can be contributed to the abnormal mitochondrial phenotype and signaling pathways.

## Discussion

To date, significant progress has been made in exploring the molecular basis and signaling pathways of CCM3-related pathogenesis of CCM, including identifying of the relevance of cell adhesion and cell-to-cell junctions, cell proliferation, cytoskeleton dynamics, angiogenesis, ROS, EndMT, and autophagy [11, 31–34]. However, the mechanisms implicated in CCM are often considered separately based on a particular experimental condition, sometimes leading to opposite conclusions. Thus far, no study undertook the critical task of organizing and prioritizing known signaling pathways to provide a definitive conclusion regarding the function of CCM3 in CCM. To explore the overall changes of mRNA expression in endothelial cells after CCM3 knockout, we utilized RNA-sequencing to compare endothelial cells of brain microvessels obtained from WT and *Ccm3*^ECKO^ mice. The accumulated data demonstrated that the knockout of CCM3 in mice has a considerable impact on mitochondrial oxidative phosphorylation pathway, in addition to previously documented effects on angiogenesis and cell adhesion. Our RNA sequencing data suggest that CCM3 protein may maintain the cellular homeostasis of ROS by affecting the structure and function of mitochondria. Although the levels of ETC proteins were not changed significantly in human endothelial cells after the treatment with siCCM3, the expression of their genes was decreased, indicating that siCCM3 impacts the mRNA level of all components of ETC. Additionally, qPCR and Western Blotting revealed that the expression of ATPIF1 was changed significantly after CCM3 knockout, and overexpression of ATPIF1 neutralized some of the signaling changes caused by siCCM3. These findings suggest that ATPIF1 can be a modulator in the CCM3 pathway. Interestingly, ATPIF1 was also present in the metabolic and energy pathway and was the only gene not belonging to the ETC. Based on these results, we concluded that ATPIF1 could constitute a link between energy metabolism and angiogenesis.

Angiogenesis is the process of the development of new blood vessels from the previously existing vascular network. Angiogenesis involves endothelial cells proliferation, differentiation, migration, adhesion, and signaling [35, 36]. We determined the signaling changes in HUVECs after the treatment with siCCM3 or/and oeATPIF1 by Western Blotting and demonstrated that oeATPIF1 treatment could rescue, at least in part, certain signaling pathways, such as p-MLC2 and p-Smad2. Additionally, we found that cell proliferation was increased, cell-to-cell junctions were disrupted, and cell migration was increased after siCCM3 treatment, and these effects could be neutralized by oeATPIF1. The absence of significant changes in tube formation after the administration of siCCM3 or oeATPIF1 could be explained by the fact that tube formation involves the combination of cell adhesion, migration, proliferation, and microenvironmental factors [12]. Thus, ATPIF1 attenuates certain angiogenic activities, such as cell proliferation, cell adhesion, cell migration, and signaling, induced by the loss of CCM3. To further explore the mechanism underlying these phenomena, we first hypothesized that it was related to mitochondrial function. This notion was based on the results of RNA sequencing related to the changes in mitochondrial energy metabolism. Subsequently, we found that the combination of siCCM3 and oeATPIF1 significantly preserved ATP concentration in mitochondria. But the oxygen consumption rate and spare respiratory capacity were decreased, indicating that the oeATPIF1 treatment compromised mitochondrial oxidative phosphorylation. The regulation of mitochondrial function by ATPIF1 can be contradictory, as ATPIF1 increases the number of cristate and promotes the maintenance of the elongated morphology of mitochondria [21, 22], which means increased mitochondrial function with oeATPIF1, and the effect of ATPIF1 on the morphology of mitochondria was also confirmed by our data. But other studies suggested that ATPIF1 inhibits the ATP synthase activity, so does our seahorse data. Additionally, we observed that siCCM3 treatment destroys the morphology of mitochondria, and the immunofluorescence images of CCM3-KO cells confirmed the dramatic impact of long-term loss of CCM3 on mitochondrial architecture. Although the Western Blotting results of CCM3-related signaling pathway markers in selected cells didn’t resemble the siRNA treated cells, the difference in signaling pathways between CRISPR-knockout cells and siRNA-transfected cells has been demonstrated in earlier studies [37, 38]. The compromised mitochondrial membrane potential in CRISPR-CCM3 EPCs was also in line with the changes in morphology. Interestingly, s-oeATPIF1 treatment did not restore the ΔΨm after FCCP treatment, most likely due to the inhibition of the ATPase activity of ATP synthase by ATPIF1. Thus, ATPIF1 may protect cells from ΔΨm-related generation of ROS [39]. Moreover, s-oeATPIF1 inhibited PINK1 activation which was caused by CCM3-KO. These results suggest that the short- or long-term loss of CCM3 destroys mitochondria, while overexpression of ATPIF1 preserves the concentration of ATP and maintains the normal elongated morphology of mitochondria.

The morphology changes in CCM3-KO cells were more severe than siCCM3 cells. Moreover, the alterations in signaling pathways of KLF4 and Tie2 also appeared to be more pronounced in CCM3-KO than in siCCM3 cells. We postulated that the duration of CCM3 loss might have contributed to these differences since the siCCM3 treatment typically lasts for 2 or 3 days, but the in *vivo* samples were collected at least 5 days after knocking out CCM3. Thus, although structural and functional changes in endothelial cells occur shortly after the loss of CCM3, the downstream manifestation will appear at a later period. In this regard, a study was published in which in vivo loss of CCM3 was considered chronic only after 30 days [40], reinforcing the notion that the siRNA treatment may not reveal the function of the gene accurately.

Interestingly, we could not successfully overexpress ATPIF1 in CCM3-KO cells, although the efficiency of s-oeATPIF1 in WT cells was extremely high. Initially, we hypothesized that this problem is related to protein degradation via the ubiquitin pathway. However, the administration of MG132 did not affect the protein level of ATPIF1 in CRISPR-CCM3 treated with s-oeATPIF1. Moreover, the level of ATPIF1 mRNA in CRISPR-CCM3 + s-oeATPIF1 cells was significantly lower than in s-oeATPIF1 cells. These results indicated that CRISPR-CCM3 regulates the transcription of *ATPIF1*. The knockout of CCM3 in s-oeATPIF1 cells (s-oeATPIF1 + CRISPR-CCM3 cells) allowed us to obtain cells with high efficiency of both CCM3 knockout and ATPIF1 overexpression. We found that the expression of both KLF4 and Tie2 was intact in s-oeATPIF1 + CRISPR-CCM3 cells, while the markers of signaling in CRISPR-CCM3 + s-oeATPIF1 were the same as in CRISPR-CCM3 cells, despite suboptimal efficiency of ATPIF1 overexpression. Given the destruction of mitochondria in CRISPR-CCM3 cells, we concluded that ATPIF1 maintains the normal structure of mitochondria in s-oeATPIF1 + CRISPR-CCM3 cells before mitochondrial damage is induced by the loss of CCM3. Under this condition, s-oeATPIF1 can maintain normal signaling pathways. Eventually, the mitochondria were destroyed by the prolonged loss of CCM3, resulting in an inhibition of ATPIF1 transcription, and changing the phenotype of s-oeATPIF1 + CRISPR-CCM3 cells to that resembling CRISPR-CCM3 cells.

KLF4 is a versatile transcription factor participating in the pathogenesis of CCM through the EndMT, MEKK3-KLF4, and VEGF pathways [13, 30, 41]. Also, KLF4 induces mitochondria biogenesis and fusion [42, 43]. However, the current data suggest that KLF4 is implicated in the destruction of mitochondria. This conclusion was reached since KLF4 was increased in CRISPR-CCM3 cells with disrupted mitochondria. We also found that KLF4 may function as the upstream regulator of other signaling pathways because, in contrast to Tie2, its expression was normalized after re-overexpressing CCM3 for 2 days. The expression of Tie2 was significantly down-regulated only after 5 days of CCM3 re-overexpression.

## Conclusions

Our data suggest that the loss of CCM3 affects intercellular junctions of endothelial cells, cell proliferation, cell migration, and the structure of mitochondria. Overexpression of ATPIF1 maintains mitochondrial structure and attenuates alterations in endothelial cell function triggered by the loss of CCM3. However, long-term loss of CCM3 destroys mitochondria completely and inhibits the expression of ATPIF1. KLF4 can function as the upstream regulator of both mitochondrial architecture and endothelial cell-mediated angiogenesis.

## Materials and methods

### ***Ccm3***^ECKO^ mice and RNA sequencing

*Ccm3*^fl/fl^ mice were crossed with *Mfsd2a-CreERT2* mice, in which CreERT2 recombinase expression is driven by the *Mfsd2a* promoter to generate *Ccm3*^fl/fl^; *Mfsd2a-CreERT2* mice.

Mouse brain microvascular endothelial cells were isolated, as described previously [10]. A total of 2 μg RNA per sample was used for RNA sequencing. After adaptor trimming and low-quality sequence filtering, the reads were mapped to the mouse reference genome version.

*Ccm3* knockout related DEGs were analyzed by Gene Ontology Biological Process (GOBP) and KEGG in the Database for Annotation, Visualization, and Integrated Discovery (DAVID) (https://david.ncifcrf.gov/) [44], and an enrichment map was built by Cytoscape with Enrichment Map Apps. Each node denotes one enriched GOBP cluster (p < 0.005, FDR q < 0.1, overlap cutoff > 0.5).

### Quantitative PCR (qRT-PCR)

Total RNA from HUVECs or EPCs was prepared using Trizol (15,596,018, Invitrogen), and the single-strand cDNA was synthesized using iScript cDNA Synthesis Kit (1,708,891, BIO-RAD). Real time PCR was performed with CFX-96 (BIO-RAD) using the iQ SYBR Green Supermix (1,708,882, BIO-RAD). Primers were inquired from https://www.origene.com/, and double checked by NCBI blast. All values were normalized with β-actin abundance. Data were presented as the average of triplicates ± SD.

### Western blotting

HUVECs and EPCs were directly lysed in 1.25 × loading buffer (62.5 mM Tris–HCl, 2.5% SDS, 12.5% Glycerol, 0.05% bromophenol blue, 125 mM DTT) with Protease Inhibitor Cocktail (11,697,498,001, Roche). Lysates were loaded onto SDS-PAGE gels and after electrophoresis transferred to 0.45 μm PVDF membrane (Millipore). After blocking (5% non-fat dried milk in Tris buffered saline (TBS) with 0.1% Tween-20), membranes were probed with first antibodies overnight at 4 °C. The membranes were then washed by TBST for 10 min × 3 times, incubated with second antibodies at room temperature for 1 h. The chemiluminescence (ECL) system and exposure to film was used to visualize the bands, taking number of different exposures.

Antibodies: CCM3/PDCD10 (Abcam, ab180706, 1:1000); OXPHOS (Abcam, ab110413, 1:1000); S100A11 (Proteintech, 10,237-1-AP, 1:1000); ATPIF1 (Proteintech, 12,067-1-AP, 1:1000);PARK7 (Santa Cruz, sc-55572, 1:500); CLIC4 (Santa Cruz, sc-135739, 1:500); β-actin (CST, 3700S, 1:2000); VEGFR2 (CST, 2479S, 1:2000); Tie2 (CST, 4224S, 1:500); p-Tie2 (CST, 4226S, 1:500); p-AKT (CST, 4060S, 1:1000); β-catenin (Santa Cruz, sc-7963, 1:2000); KLF4 (CST, 4038S, 1:500); p-Smad2 (CST, 3108S, 1:500); p-MLC2 (CST, 3674S, 1:1000); MFN1 (Abcam, ab57602, 1:1000); OPA1 (Abcam, ab42364, 1:1000); DRP1(Abcam, ab56788, 1:1000); FIS1 (Enzo, 10,121,719, 1:500); GAPDH (CST, 5174S, 1:5000).

### Cell culture and treatment

HUVECs and EPCs were obtained from Yale VBT core, Yale School of Medicine and grown in EGM-2 BulletKit (CC-3162, Lonza). For the siRNA treatment assay, cells were cultured at 60–70% confluence in six-well plates and transferred with total 30 pmol siCCM3 (sc-62084, Santa Cruz), siATPIF1 (sc-78711, Santa Cruz), siS100A11 (sc-60314, Santa Cruz) or siNC (sc-37007, Santa Cruz) using Lipofectamine RNAiMAX Transfection Reagent (13,778,150, Invitrogen), according to the manufacture’s instruction. Cells were cultured 48 h before harvest. For the MG132 treatment, cells were incubated with 10 μm MG132 or the same volume of DMSO as negative control for 12 h before harvest.

For EdU staining, siRNA and pLEX-ATPIF1 treated HUVECs were stained with EdU (Click-iT® EdU Imaging Kits, C10337) according to the manufacture’s instruction.

### Plasmids and lentivirus

The LentiCRISPRv2 plasmid was used to construct CRISPR-CCM3 plasmid according to the manufacture’s instruction. Two sgCCM3 were selected after verification the effect of knockout by Western Blotting, sgCCM3 Forward 1: CACCGGAGCCGCTTTCATCAAGGTG; Reverse 1: AAACCACCTTGATGAAAGCGGCTCC; Forward 2: CACCGGCTGATGATGTAGAAGGTAA, Reverse 2: AAACTTACCTTCTACATCATCAGCC. CRISPR-CCM3 virus were amplified in HEK 293 T cells and purified by 0.45 μm filter. The LentiORF pLEX-MCS Vector (Thermo Scientific) was used to construct oeATPIF1 according to the manufacture’s instruction. ATPIF1 CDS was amplified by the primer, Forward: TTGCGGCCGC ATGGCAGTGACGGCGTTGGCGG, Reverse: GACGCGT TTAATCATCATGTTTTAGCATTTTGA. oeATPIF1 virus were amplified in HEK 293 T cells and purified by 0.45 μm filter. Cells were cultured to 60% confluence, then change the normal medium to mixed medium with normal medium: lentivirus = 1:1 for 12 h, cells were selected with 1 μg/mL puromycin when the negative cells were all killed by puromycin, this process usually lasts 2–3 days.

### Immunofluorescence confocal microscopy

HUVECs, HDMECs and EPCs were fixed in 4% PFA for 20 min, washed with PBS and permeabilized with 0.1% Triton X-100 for 5 min. After blocking with 5% donkey serum for 30 min, slides were incubated with primary antibodies at 4 °C overnight, and then were washed with PBS three times and incubated with secondary antibodies (1:200–1:400) at room temperature for 1 h. After washing in PBS three more times, slides were mounted with VECTASHIELD Mounting medium with DAPI (Vector Laboratory) and photographed. Confocal images were taken with a Leica SP5 microscope and analyzed with ImageJ software.

Immunofluorescence antibodies: ZO-1 (Invitrogen, 61–7300, 1:100), VE-Cadherin (Santa Cruz, sc9989, 1:100), ATPIF1 (Proteintech, 12,067-1-AP, 1:100), T-FAM (CST, 8076S, 1:100), MFN1 (Abcam, ab57602, 1:100), FIS1 (Proteintech, 10,956-1-AP, 1:100).

### Endothelial cell monolayer migration

After infected, the endothelial cells were subjected to a scratch wound injury. Cells were plated with fresh media and were further cultured for 16 h. Endothelial cell migration in culture was determined by measuring “wound” areas in cell monolayers. Three different images from each well along the wound were captured by a digital camera under a microscope (at a magnification of 4x). Wound area was measured and analyzed by ImageJ.

### Endothelial cell tube formation in Matrigel

Twenty-four-well tissue culture plates were coated with 0.2 ml of Matrigel (Corning) per well and incubated at 37 °C for 1 h (36). After infected, HUVECs were resuspended and plated on Matrigel (0.2 ml) in the 24-well plate for 16 h. The area covered by branched cells was measured and analyzed by ImageJ.

### Mitochondrial function assays

Mitochondrial ATP concentration: Mitochondria in HUVECs was isolated using Mitochondria Isolation Kit for Cultured Cells (Thermo Scientific) according manufacture’s instruction, and the ATP concentration of isolated mitochondria was measured by ATP Determination Kit (Thermo Scientific).

Mitochondrial metabolism by seahorse XF96: After treated, HUVECs cells were resuspended and cultured in XF 96-well culture microplate with 20,000 cells in 80 ml medium, then incubated them overnight at 37 °C in 5% CO_2_. At the same time, prepare the cartridge overnight. The mito-stress assay was composed of 1 μM Oligomycin, 1 μM FCCP and 1 μM Rotenone/Antimycin in seahorse XF base medium with 10 mM glucose, 1 mM pyruvate and 2 mM L-Glutamine. Oxygen consumption rates (OCR) were measured using an XF96 Extracellular Flux Analyzer.

Mitochondrial membrane potential: The ΔΨm of EPCs were analyzed by MitoTracker Red CM-H2XRos kit (Invitrogen, M7513) according manufacture’s instruction. Cells were treated with DMSO or FCCP (Sigma, C2920) (10 μM) for 2 h.

### Statistical analysis

All data are presented as means ± SEM. Statistical differences were measured by either two-tailed Student’s t test or one- or two-way ANOVA with Bonferroni post-test. Data analyses were performed using Graph Pad Prism software version 8.0. A value of *p* < 0.05 was considered statistically significant.

## Supplementary Information

**Additional file 1: Fig. S1. ** Clustering Analysis for CCM3^ECKO^ Cell RNA-Seq Result. Total 92 genes with significant differential expression were subjected to DAVID GOBP analysis, and an enrichment map was built by Cytoscape with Enrichment Map Apps. Each node denotes one enriched GOBP cluster (p < 0.005, FDR q < 0.1, overlap cutoff > 0.5). Node size is proportional to the total number of genes in each cluster; Edge width is proportional to the number of shared genes in each cluster. Similar GOBP clusters were sorted and marked with circles and labels.

**Additional file 2: Fig. S2. **OXPHOS and glycolysis related genes in siRNA or lentivirus treatment endothelial cells. **a** glycolysis related gene in HUVECs after siCCM3 treatment. **B, c **OXPHOS related genes in selected CRISPR-CCM3 (**b**) and s-oeATPIF1 (**c**) EPCs. **d**, **e **glycolysis related genes in selected CRISPR-CCM3 (**d**) and s-oeATPIF1 (**e**) EPCs.

**Additional file 3: Fig. S3. **Confirm the efficiency of lentivirus. CRISPR-CCM3 (**a**) and s-oeTPIF1 (**b**) was confirmed by qRT-PCR and western blotting. **c **the efficiency of CRISPR-CCM3 and overexpression of CCM3 in EPCs examined by immunofluorescence.

## Data Availability

The datasets used and/or analyzed during the current study are available from the corresponding author on reasonable request.

## Abbreviations

CCM: Cerebral cavernous malformation
ATPIF1: ATPase inhibitory factor 1
HUVEC: Human umbilical vein endothelial cells
EPC: Endothelial progenitor cells
ETC: Electron transport chain
GOBP: Gene Ontology Biological Process
p-MLC: Phospho-myosin light chain
EndMT: Endothelial to mesenchymal transition
KLF4: Krüppel-like factor 4
S100A11: S100 calcium binding protein A11
CLIC4: Chloride intracellular channel 4
PARK7: Parkinsonism associated deglycase
MFN1: Mitofusin 1
OPA1: Optic atrophy 1
DRP1: Dynamin related protein 1
FIS1: Fission, mitochondrial 1
PINK1: PTEN induced putative kinase 1
