# Supplementary material for: ATPIF1 maintains normal mitochondrial structure which is impaired by CCM3 deficiency in endothelial cells
Source: Cell Biosci. 2021 Jan 9;11:11. doi: 10.1186/s13578-020-00514-z (PMC7796565; doi:10.1186/s13578-020-00514-z)
Supplement: Supplementary file 1 — Additional file 1: Fig. S1. Clustering Analysis for CCM3ECKO Cell RNA-Seq Result. Total 92 genes with significant differential expression were subjected to DAVID GOBP analysis, and an enrichment map was built by Cytoscape with Enrichment Map Apps. Each node denotes one enriched GOBP cluster (p < 0.005, FDR q < 0.1, overlap cutoff > 0.5). Node size is proportional to the total number of genes in each cluster; Edge width is proportional to the number of shared genes in each cluster. Similar GOBP clusters were sorted and marked with circles and labels. [file 13578_2020_514_MOESM1_ESM.docx]

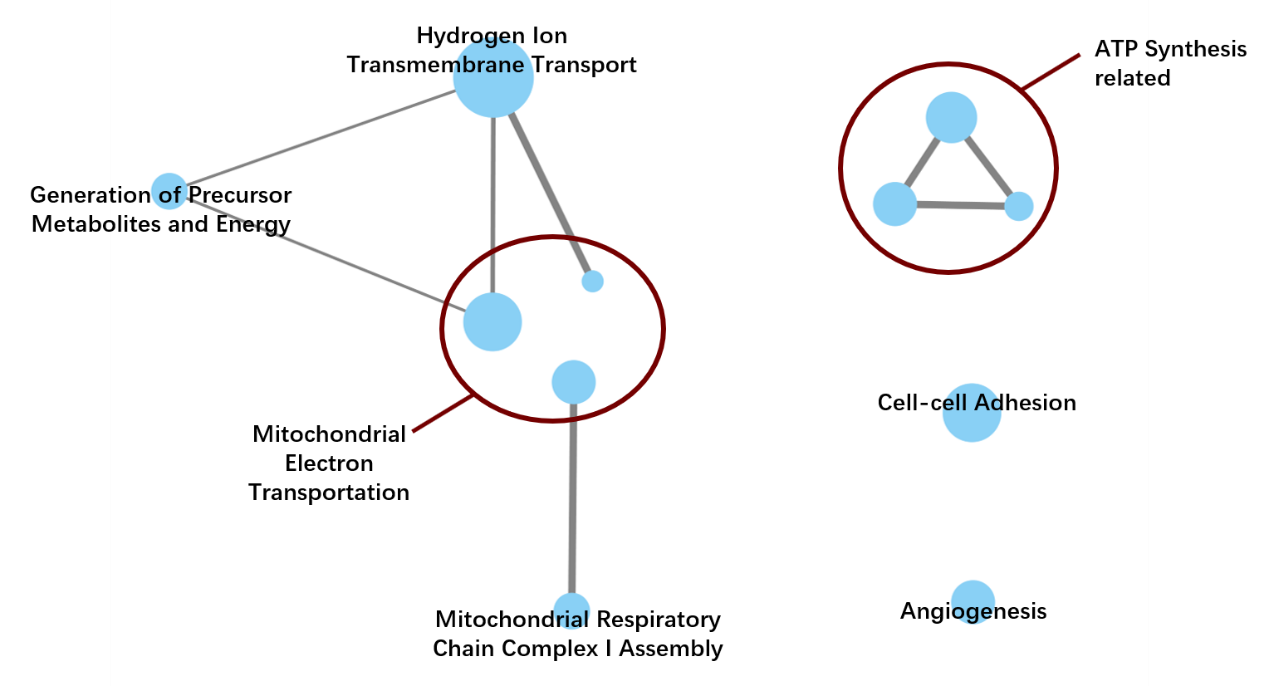


**Fig. S1** Clustering Analysis for CCM3^ECKO^ Cell RNA-Seq Result. Total 92 genes with significant differential expression were subjected to DAVID GOBP analysis, and an enrichment map was built by Cytoscape with Enrichment Map Apps. Each node denotes one enriched GOBP cluster (p < 0.005, FDR q < 0.1, overlap cutoff > 0.5). Node size is proportional to the total number of genes in each cluster; Edge width is proportional to the number of shared genes in each cluster. Similar GOBP clusters were sorted and marked with circles and labels.
