# Supplementary material for: ATPIF1 maintains normal mitochondrial structure which is impaired by CCM3 deficiency in endothelial cells
Source: Cell Biosci. 2021 Jan 9;11:11. doi: 10.1186/s13578-020-00514-z (PMC7796565; doi:10.1186/s13578-020-00514-z)
Supplement: Supplementary file 2 — Additional file 2: Fig. S2. OXPHOS and glycolysis related genes in siRNA or lentivirus treatment endothelial cells. a glycolysis related gene in HUVECs after siCCM3 treatment. B, c OXPHOS related genes in selected CRISPR-CCM3 (b) and s-oeATPIF1 (c) EPCs. d, e glycolysis related genes in selected CRISPR-CCM3 (d) and s-oeATPIF1 (e) EPCs. [file 13578_2020_514_MOESM2_ESM.docx]

**Fig.S2** OXPHOS and glycolysis related genes in siRNA or lentivirus treatment endothelial cells. **a** glycolysis related gene in HUVECs after siCCM3 treatment. **b-c** OXPHOS related genes in selected CRISPR-CCM3 (**b**) and s-oeATPIF1 (**c**) EPCs. **d-e** glycolysis related genes in selected CRISPR-CCM3 (**d**) and s-oeATPIF1 (**e**) EPCs.
