# Supplementary material for: ATPIF1 maintains normal mitochondrial structure which is impaired by CCM3 deficiency in endothelial cells
Source: Cell Biosci. 2021 Jan 9;11:11. doi: 10.1186/s13578-020-00514-z (PMC7796565; doi:10.1186/s13578-020-00514-z)
Supplement: Supplementary file 3 — Additional file 3: Fig. S3. Confirm the efficiency of lentivirus. CRISPR-CCM3 (a) and s-oeTPIF1 (b) was confirmed by qRT-PCR and western blotting. c the efficiency of CRISPR-CCM3 and overexpression of CCM3 in EPCs examined by immunofluorescence. [file 13578_2020_514_MOESM3_ESM.docx]

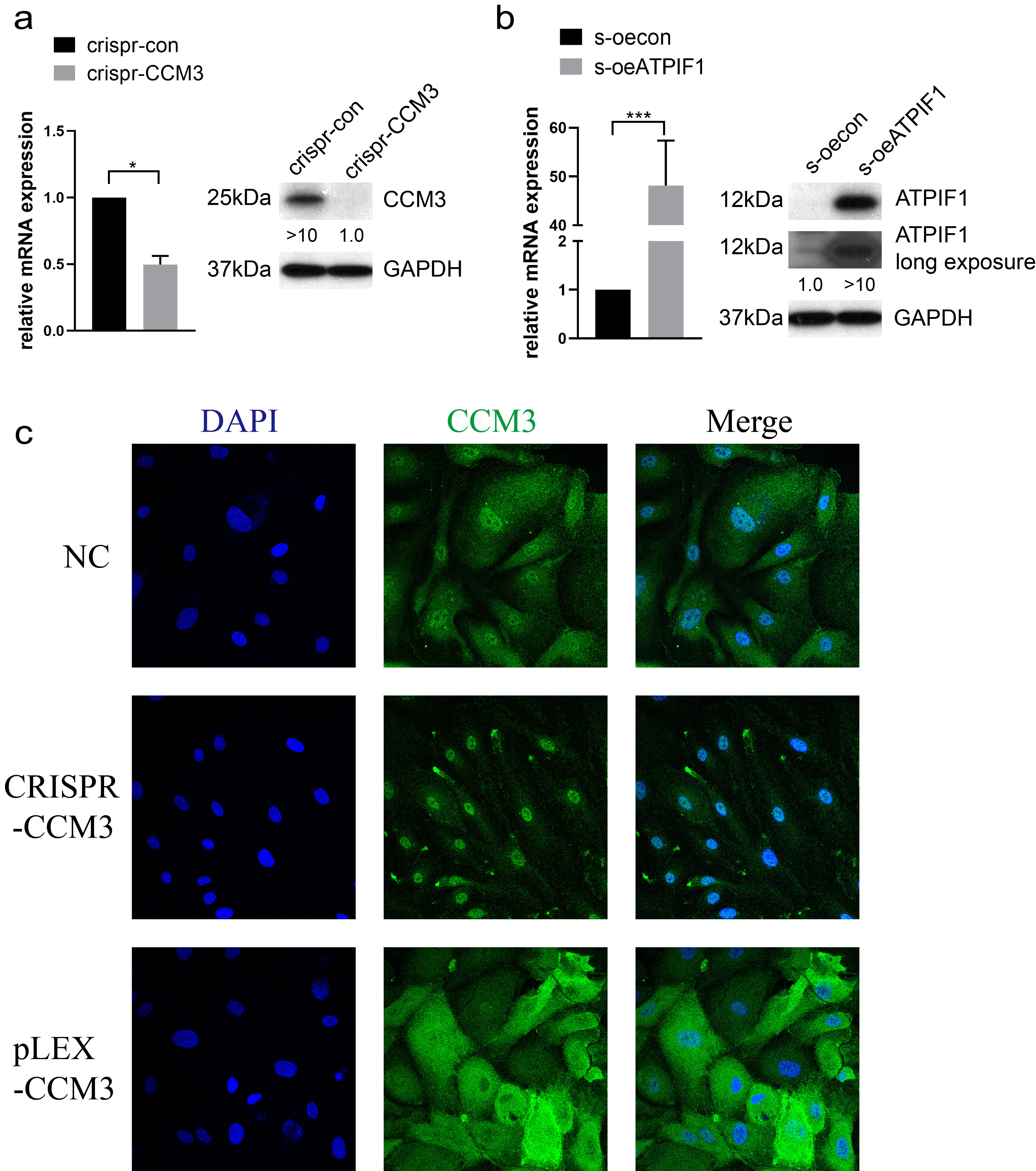


**Fig.S3** confirm the efficiency of lentivirus. CRISPR-CCM3 (**a**) and s-oeTPIF1 (**b**) was confirmed by qRT-PCR and western blotting. **c** the efficiency of CRISPR-CCM3 and overexpression of CCM3 in EPCs examined by immunofluorescence.
